# Supplementary material for: Structural and Functional Effects of the Interaction Between an Antimicrobial Peptide and Its Analogs with Model Bacterial and Erythrocyte Membranes
Source: Biomolecules. 2025 Aug 7;15(8):1143. doi: 10.3390/biom15081143 (PMC12383913; doi:10.3390/biom15081143)
Supplement: Supplementary file 1 [file biomolecules-15-01143-s001.zip › biomolecules-3739848-Supplementary Materials.pdf]

SUPPLEMENTARY MATERIAL

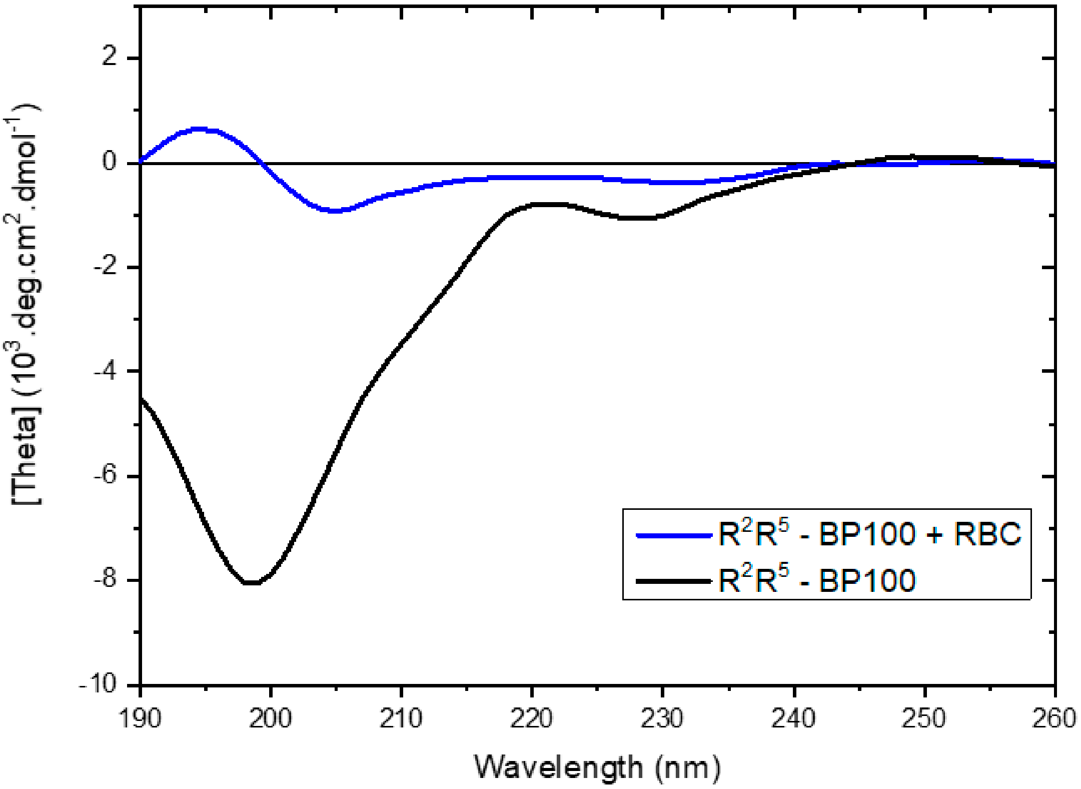

Figure S1. CD spectra of R^2R^5-BP100 in 10 mM Tris-HCl pH 7.4 buffer (black line), and in RBC LUVs (blue line). R^2R^5-BP100 was 200 μM and LUVs concentration was 1.00 mM. The cell pathlength was 0.1 mm and the cell volume was 0.3 mL.
